# Opalski Syndrome: An Intricate Case of Lateral Medullary Infarction With Unconventional Neurological Features and Diagnostic Challenges

**DOI:** 10.7759/cureus.66947

**Published:** 2024-08-15

**Authors:** Vaibhav, Rohit Sharma, Aditya Duhan, Vipul Kaliraman, Diksha Yadav

**Affiliations:** 1 Department of Neurology, Pt. Pandit Bhagwat Dayal Sharma University of Health Sciences, Rohtak, IND; 2 Department of Medicine, Pt. Pandit Bhagwat Dayal Sharma University of Health Sciences, Rohtak, IND; 3 Department of Radiodiagnosis, Pt. Pandit Bhagwat Dayal Sharma University of Health Sciences, Rohtak, IND; 4 Department of Medicine, Maulana Azad Medical College, New Delhi, IND

**Keywords:** acute ischemic stroke, hemianesthesia, medulla oblongata infarction, ipsilateral hemiparesis, lateral medullary syndrome, wallenberg syndrome, opalski syndrome

## Abstract

Opalski syndrome is a rare variant of Wallenberg syndrome characterized by ipsilateral hemiparesis. We present a case involving a 50-year-old male who exhibited symptoms of right-sided upper and lower limb weakness, dizziness, and ataxia. T2-weighted magnetic resonance imaging (MRI) identified an infarction in the right lateral medulla. This case displayed several unique and atypical features, including the negative Babinski sign, absence of dysphagia, and dysarthria. The diagnosis was confirmed through meticulous clinical examination and advanced radiological investigations, such as MRI. This case report underscores the critical importance of thorough clinical evaluation and the correlation of findings with radiological investigations to reduce morbidity and mortality in patients with Opalski syndrome.

## Introduction

Opalski syndrome is an uncommon variant of Wallenberg syndrome, also termed lateral medullary syndrome (LMS). Wallenberg syndrome is characterized by a distinct set of symptoms resulting from an infarction in the lateral segment of the medulla oblongata, located in the lower brainstem [1-3]. These clinical manifestations include vertigo, diplopia, dysarthria, Horner's syndrome, and sensory deficits involving the ipsilateral face and contralateral limbs [1-4]. Opalski syndrome is specifically differentiated by the presence of ipsilateral hemiparesis (muscle weakness on the same side as the lesion) in addition to the classic symptoms of Wallenberg syndrome [1-3].

LMS is a vascular syndrome of the posterior cerebral circulation. The key distinguishing feature of Opalski syndrome is ipsilateral hemiparesis or hemiplegia, which results from the extension of the lateral medullary infarct to involve the corticospinal fibers caudal to the pyramidal decussation. This differentiates it from other variants of the LMS.

We hereby present a case of Opalski syndrome with varied presentations. This case report aims to describe the diagnostic and management challenges associated with this rare variant of the LMS. The presentation of this case contributes to the medical literature by increasing awareness of the occurrence and varied manifestations of this uncommon condition across many global regions.

## Case presentation

A 50-year-old male with a history of chronic smoking presented to our department with complaints of weakness in his right upper and lower limbs, dizziness, ptosis of the right eye, and ataxia for the last two days. The patient experienced sudden-onset dizziness upon rising from a chair, which was exacerbated by bending his neck to the right. He also developed ataxia that was sudden, constant, and non-progressive, accompanied by a tendency to sway to the right side of his body.

The patient also gave a history of ptosis of the right eyelid since the onset of the illness, which was sudden in onset, non-progressive, and without diurnal variation. There was an associated history of hiccups since the day of the presentation, which resolved spontaneously on the same day. There was no history of fever or headache. The patient denied any history of dysphagia, slurred speech, loss of bowel or bladder control, loss of consciousness, altered taste or smell sensation, or facial deviation.

In addition, there was a history of herpes zoster, one year back, involving the skin below the left upper abdomen that persisted for five months. The patient also had a 15-year history of hypertension, for which he had been on irregular treatment. Thirty years prior, the patient had been involved in a roadside accident, resulting in chronic back pain since then.

On examination, the patient was conscious, cooperative, and oriented. Vital signs were within normal limits: blood pressure was 124/72 mmHg, pulse rate was 96 beats per minute, SpO_2_ was 97% on room air, and respiratory rate was 20 breaths per minute.

On neurological examination, cranial nerve examination revealed ptosis of the right eye, with other findings including the size of pupils and light reflex being within normal limits. The motor system examination indicated normal muscle bulk except for atrophy of the right thenar muscles. Muscle tone was normal in the left upper and lower limb, but was decreased in the right upper limb and increased in the right lower limb. Muscle strength was 4/5 in the right upper and lower limbs, while it was normal in the left upper and lower limbs. Deep tendon reflexes were intact and plantar reflexes were flexor bilaterally. Sensory examination revealed hemianesthesia to pain and temperature on the left side, while other sensations such as vibration, proprioception, and two-point discrimination were intact. The patient exhibited a drunken gait during ambulation.

**Figure 1:**
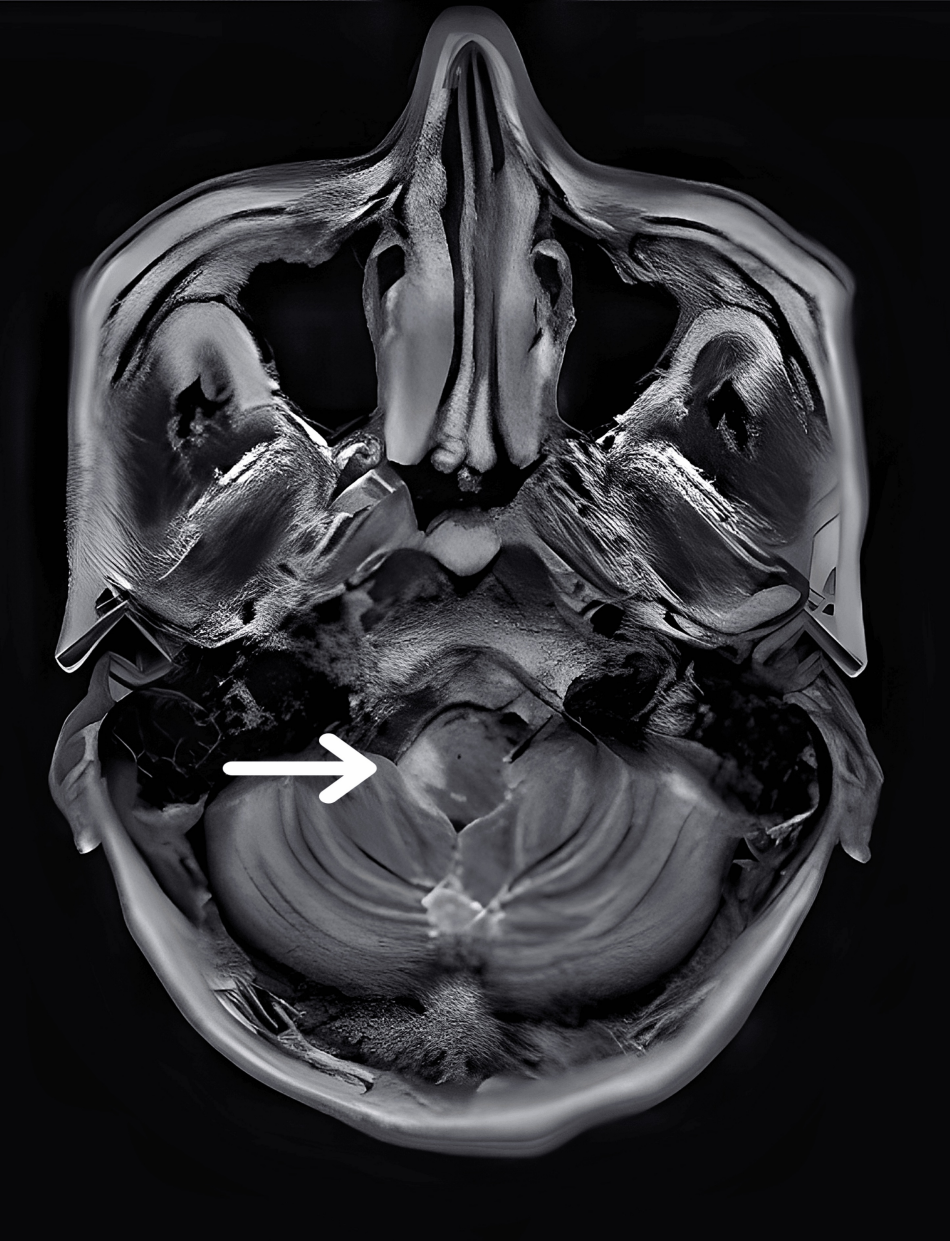
T2-weighted magnetic resonance imaging (MRI) demonstrating an infarction in the right lateral medulla

Based on the clinical evaluation, we suspected right-sided LMS with right hemiplegia (upper motor neuron). Routine blood investigations and urine analyses were within normal limits. Blood glucose levels, lipid profile, liver function tests, and renal function tests were also within normal limits. T2-weighted magnetic resonance imaging (MRI) revealed an infarction in the right lateral medulla (Figure 1).

The patient was administered aspirin 75 mg once daily (OD), clopidogrel 75 mg OD, atorvastatin 20 mg OD, pantoprazole 40 mg OD before breakfast (BBF), betahistine 8 mg three times daily (TDS), folic acid 5 mg OD, and a combination of gabapentin 200 mg with nortriptyline 10 mg OD.

## Discussion

The presented case of lateral medullary infarction with ipsilateral hemiparesis was diagnosed as Opalski syndrome, which exhibited unique characteristics such as a negative Babinski sign, absence of dysphagia, and dysarthria. The diagnosis was confirmed through meticulous clinical examination and advanced radiological investigations, including MRI.

This case report elucidates the presentation of LMS on the MRI brain, in conjunction with clinical findings specific to Opalski syndrome. In contrast to numerous cases demonstrating ipsilateral corticospinal deficits in LMS with a positive Babinski sign and contralateral sensory deficits [5,6], our case exhibited a negative Babinski sign, consistent with findings from other studies [1,7,8]. In a comprehensive study of LMS involving 130 patients, seven cases were identified with mild ipsilesional hemiparesis without reflex abnormalities or Babinski's sign [7]. The absence of a positive Babinski sign in Opalski syndrome is atypical and may suggest limited involvement of the pyramidal tract. However, the presence of hyperreflexia and ipsilateral hemiparesis supports the diagnosis of Opalski syndrome [1].

The primary etiology of Opalski syndrome is an infarction in the lateral medulla, caudal to the pyramidal decussation, typically resulting from vertebral artery pathology or anatomical variations. This leads to the characteristic clinical presentation of LMS accompanied by ipsilateral hemiparesis [1,8,9].

There is considerable variability in sensory involvement reported in Opalski syndrome across various case reports [1,2,4,5-8,10-12]. The absence of dysphasia and dysarthria in our case is another notable feature, differing from several other reported cases [1,8,10,11,13].

The predominant cause of Opalski syndrome is ischemic stroke, often associated with vertebral artery dissection, stenosis, occlusion, or atherosclerotic disease. The critical feature is the extension of the infarct from the lateral medulla into the upper cervical spinal cord, resulting in the characteristic ipsilateral hemiparesis.

In LMS, compromise of the posterior inferior cerebellar artery (PICA) leads to lateral medullary infarction. LMS is frequently referred to as PICA syndrome due to the predominance of atherothrombotic occlusion of the vertebral artery as the leading cause of LMS. Vertebral artery disease involving PICA is the most common etiology of LMS (67%) [14].

The extent of lateral medullary damage influences the combination and severity of LMS presentations, which can vary significantly among patients. Understanding the specific symptoms and signs associated with different levels of medullary damage is essential for accurate diagnosis and effective management of LMS [15,4].

A thorough neurological examination, including assessment of reflexes and sensory deficits, along with neuroimaging findings, is crucial for distinguishing Opalski syndrome from other similar conditions, such as Babinski-Nageotte syndrome. Opalski syndrome is characterized by lateral medullary and upper cervical cord infarction with ipsilateral hemiparesis, whereas Babinski-Nageotte syndrome involves both medial and lateral medullary infarction with contralateral hemiparesis. In Opalski syndrome, the corticospinal fibers are affected post-decussation in the medulla, resulting in ipsilateral hemiparesis. In Babinski-Nageotte syndrome, the pyramidal tract is affected pre-decussation, leading to contralateral hemiparesis.

For patients with LMS, the mainstays of treatment include antithrombotic therapy, statins, physiotherapy, and other rehabilitative measures such as occupational therapy and speech therapy. An interdisciplinary approach is essential to achieve favorable health outcomes in LMS patients [13]. In addition, managing modifiable risk factors such as hypertension, diabetes, and smoking is of paramount importance.

## Conclusions

Opalski syndrome is an uncommon variant of lateral medullary infarction that necessitates prompt recognition and management as an acute ischemic stroke. The extent of damage to the lateral medulla significantly influences the clinical presentation, with ipsilateral hemiparesis serving as a key distinguishing feature compared to the typical Wallenberg syndrome. A high index of suspicion, coupled with prompt diagnosis and appropriate management, is essential for optimal patient outcomes in cases of Opalski syndrome.

To further improve clinical practice, it is recommended that clinicians consider Opalski syndrome in the differential diagnosis of lateral medullary infarction, especially when ipsilateral hemiparesis is present. Early use of advanced neuroimaging, such as MRI, is crucial for accurate diagnosis. In addition, clinicians should be aware of the potential for atypical presentations, which may necessitate a tailored approach to management.

## References

[1] Pandey S, Batla A (2013). Opalski's syndrome: a rare variant of lateral medullary syndrome. J Neurosci Rural Pract.

[2] Khan U, Ahmad B, Aslam A, Muhammad A, Iqbal J (2023). Opalski syndrome, a rare variant of wallenberg syndrome, the first case reported from Pakistan: a case report. Heliyon.

[3] Saleem F, Das JM (2024). Lateral medullary syndrome. StatPearls [Internet].

[4] Thapliyal K, Garg A, Singh VP (2022). Lateral medullary syndrome: case report and review of literature. J Family Med Prim Care.

[5] Kim HY, Koh SH, Lee KY, Lee YJ, Kim SH, Kim J, Kim HT (2006). Opalski's syndrome with cerebellar infarction. J Clin Neurol.

[6] Madhav S, Mithun P, George D, Bhat M, Kumar SKG and Kumar JP (2022). Opalski syndrome: a rare presentation of Wallenberg syndrome. Int J Adv Res Med.

[7] Kim JS (2003). Pure lateral medullary infarction: clinical-radiological correlation of 130 acute, consecutive patients. Brain.

[8] Ahmed Ibrahim A, Bakir A, Osman Sidow N, Mohamed Ali A, Farah Osman M, Ahmed A, Sheikh Hassan M (2023). Lateral medullary syndrome: uncommon form of brainstem stroke. Ann Med Surg (Lond).

[9] Love BB, Biller J Chapter 22 - neurovascular system. Textbook of Clinical Neurology (Third Edition).

[10] Lim W, Breitling M, Nugent B, Sinha A, Diaz K (2021). A case of medullary infarct causing central alveolar hypoventilation. Cureus.

[11] Zhang SQ, Liu MY, Wan B, Zheng HM (2008). Contralateral body half hypalgesia in a patient with lateral medullary infarction: atypical Wallenberg syndrome. Eur Neurol.

[12] Joseph AT, Toluie A, Hrehorovich PA (2024). Opalski syndrome and elucidation of lateral medullary syndrome. Cureus.

[13] Herson AB, Falk JD, Phrathep DD, Igbonagwam CB, Fischer ST, Miller BT, Leary D (2023). The value of interdisciplinary collaboration in lateral medullary syndrome rehabilitation: a case report. Cureus.

[14] Battel I, Koch I, Biddau F (2017). Efficacy of botulinum toxin type-A and swallowing treatment for oropharyngeal dysphagia recovery in a patient with lateral medullary syndrome. Eur J Phys Rehabil Med.

[15] Day GS, Swartz RH, Chenkin J, Shamji AI, Frost DW (2014). Lateral medullary syndrome: a diagnostic approach illustrated through case presentation and literature review. CJEM.

